# 7-Methoxy-4-methylcoumarin: Standard Molar Enthalpy of Formation Prediction in the Gas Phase Using Machine Learning and Its Comparison to the Experimental Data

**DOI:** 10.1021/acsomega.3c06756

**Published:** 2023-12-14

**Authors:** Fausto Díaz-Sánchez, Miguel Angel García-Castro, María Patricia Amador-Ramírez, Diego Espinosa-Morales, Jenaro Leocadio Varela-Caselis

**Affiliations:** † Facultad de Ingeniería Química de la Benemérita Universidad Autónoma de Puebla, 18 Sur y Av. San Claudio, C.P., Puebla Pue 72570, Mexico; ‡ Facultad de Ciencias Químicas de la Benemérita Universidad Autónoma de Puebla, 14 Sur y Av. San Claudio, C.P., Puebla Pue 72570, Mexico; § Dirección de Innovación y Transferencia de Conocimiento de la Benemérita Universidad Autónoma de Puebla, Prolongación de la 24 Sur y Av. San Claudio Ciudad Universitaria, C.P., Puebla Pue 72570, Mexico

## Abstract

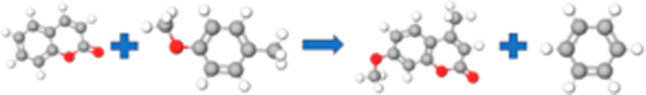

Experimentally, the standard molar enthalpy of formation in the crystalline phase at 298.15 K, Δ_f_*H*_m_°(cr) for 7-methoxy-4-methylcoumarin (7M4MC) was calculated by traditional linear regression, which was obtained by combustion calorimetry. Similarly, the standard molar enthalpy of sublimation was determined through the standard molar enthalpy of fusion and by the standard molar enthalpy of vaporization, from differential scanning calorimetry and thermogravimetry, respectively; lately using these results, the standard molar enthalpy of formation in the gas phase was calculated at 298.15 K, Δ_f_*H*_m_°(g). In addition ML was used to predict the standard molar enthalpy of formation in the gas phase for the 7M4MC, constructing an experimental data set containing three kinds of functional groups: esters, coumarins, and aromatic compounds. The procedure was performed by using multiple linear regression algorithms and stochastic gradient descent with a *R*^2^ of 0.99. The obtained models were used to compare those predicted values versus experimental for coumarins, resulting in an average error rate of 9.0%. Likewise, four homodesmic reactions were proposed and predicted with the multiple linear regression algorithm of ML obtaining good results.

## Introduction

Coumarins are heterocyclic compounds containing a lactone group. These compounds represent a wide range of natural, pharmaceutical, and phytochemical products. The interest in natural coumarins has significantly increased over time, leading to their discovery in plant species with different chemical structures and phases (crystalline and gas). In addition, many coumarins, such as 7-methoxy-4-methylcoumarin, have shown histamine release inhibition from mast cells and moderate adrenergic activity,^1^ as well as a focusing applications in lymphatic vasculature chronic disease.^2−4^ Albeit, coumarins and their derivatives exhibit antimicrobial,^5,6^ anti-inflammatory,^7,8^ antispasmodic, antiviral,^9,10^ antioxidant,^11^ and enzyme inhibitor properties.^12,13^ In addition, some coumarin-based products have demonstrated excellent results as antitumor or as photochemotherapeutic agents in psoriasis treatment.^14−16^

**Figure 1:**
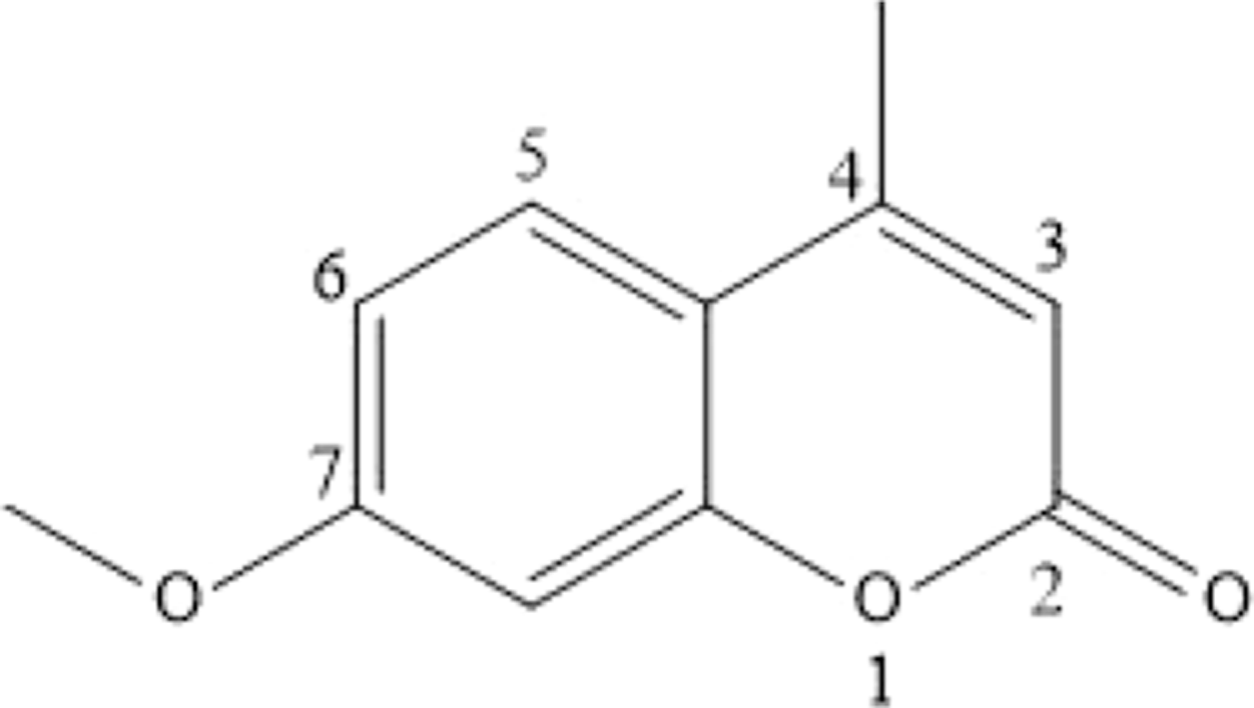
7-Methoxy-4-methylcoumarin.

Despite the multiple potential applications described above, there are very few reports on their thermochemical properties. Among these properties, one of the most important is the standard molar enthalpy of formation, which provides a better explanation and support from the synthesis process.^17,18^ Thermal and calorimetric techniques are commonly used to determine experimentally this property^19,20^ by considering the differential scanning calorimetry (DSC), thermogravimetry, and combustion calorimetry, respectively. Furthermore, it is possible to predict this property with the use of computational techniques such as machine learning algorithms.^21,22^ This work presents the experimental results related to 7-methoxy-4-methyl coumarin (7M4MC) compounds, as seen in Figure 1, which was predicted using multiple linear regression (MLR) and stochastic gradient descent regression (SGD) models in the gas phase^23,24^ based on the Benson’s group additivity method.^25^

## Experimental Section

### Materials and Purification

The 7M4MC [CAS 2555-28-4] compound (supplied by Merck), with a 0.98 mass fraction purity, was determined by DSC, although the commercial label showed 0.99. Therefore, it was purified following a recrystallization process with ethyl acetate and dried under a high vacuum for 3 h, repeating this step twice. To determine the purity, melting temperature, and fusion enthalpy, a DSC in a TA Instrument Q2000 previously calibrated for temperature and energy with high-purity indium was used. The heating rate was 3.0 °C min^–1^ under a 20.0 cm^3^ min^–1^ dry nitrogen constant flux; furthermore, a temperature range from 333.15 to 453.15 K was considered. The correspondent thermograms are shown in figure A and figure B in the Supporting Information.

Crystalline phase 7M4MC heat capacities were obtained from 273.15 to 368.15 K using a TA Instrument DSC Q2000 that was previously calibrated with aluminum oxide at a heating rate of 10.0 K min^–1^ under a nitrogen flow of 50.0 cm^3^ min^–1^. For this reference compound, the calculated heat capacity was 0.7748 J g^–1^ K^–1^ at 298.15 K reaching 99.95%, this value is closer from those reported in the literature (0.7752 J g^–1^ K^–1^ at 298.15 K).^26^

### Combustion Calorimetry

A static semimicro combustion bomb was used to determine the combustion energies. This instrument was calibrated using benzoic acid (NIST Standard Reference Material 39j) with a combustion mass energy of −(26434.0 ± 3.0) J g^–1^ (the uncertainty corresponds to expanded uncertainty), which was corrected using Coops et al. equation.^27^ The calorimetric equivalent of ϵ (calor) = (1281.2 ± 0.8) J K^–1^ (the uncertainty is twice the standard deviation of the mean) was calculated from six combustion experiments at 3.04 MPa pressure under a high purity gaseous oxygen (Air Liquide Corp., mass fraction of 0.99999) with 0.1 cm^3^ of deionized water.^28^

To maintain conditions similar to those of the reference material, the 7M4MC was oxidized considering the same parameters. The cotton-thread fuse (C_1.000_H_1.742_O_0.921_) used possess a combustion specific energy of −(16945.2 ± 4.2) J g^–1^ (the uncertainty is the standard deviation of the mean). Albeit the combustion energies in standard conditions were determined through Washburn corrections.^27^ The compounds physical properties are resumed in Table 1,^29^ where the elements’ atomic weights were those reported by IUPAC in 2021.^30^ To calculate the energy change associated with the pressure, the estimated value of (δ_u_/δ_p_)_*T*_ = −0.2 J g^–1^ MPa^–1^ at 298.15 K was used, which is a typical value considered for most of the solid organic compounds.^31^

**Table 1:** Physical Properties at *p*^o^ = 0.1 MPa

compound	*M*^a^(g mol^–^^1^)	ρ (g cm^–^^3^)	–(δ_u_/δ_p_)_*T*_ (J g^–^^1^ MPa^–^^1^)	*C*_p_(cr) (298.15 K) (J g^–^^1^ K^–^^1^)
7M4MC	190.198	1.195 ± 0.06^b^	0.200^c^	1.439 ± 0.023^d^
benzoic acid	122.123	1.320^c^	0.115^c^	1.209^c^
cotton	28.502	1.500^c^	0.289^c^	1.674^c^

a Based on the 2021 IUPAC recommendation.^30^

b Calculated using Advanced Chemistry Development (ACD/Laboratories) Software v11.02.

c Estimated value in the reference at *T* = 298.15 K.^31^

d Experimental average value from two experiments using a DSC device. Its uncertainty corresponds to expanded uncertainty with a level of confidence of approximately 95%. Including the contributions from the calibration and *u*(*T*) = 0.1 K. The experiments were realized under average atmospheric pressure (78.8 kPa), *u*(*p*) = 1 kPa.

### Thermogravimetry

The indirect method of thermal gravimetric analysis (TGA) was used to determine the vaporization enthalpy using the Langmuir equation.
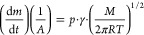
1where (d*m*/d*t*) is the rate of mass loss, *A* is the area which was subjected to the vaporization process, *T* is the temperature, *p* is the vapor pressure, *R* is the ideal gas constant, *M* is the molar mass of the compound, and γ is a vaporization constant.

Combining Clausius–Clapeyron’s to eq 1 yielded the expression that is applied to calculate the enthalpy of vaporization
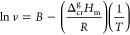
2where  and *B* includes the integration constant and the term . Using eq 2, it was possible to obtain the vaporization enthalpies by applying a linear adjustment to ln *v* vs 1/*T*. A TA Instruments Q500 device, previously calibrated for mass and temperature, was used to register the term d*m*/d*t* with high precision. The thermogravimetric system was tested with phenanthrene and pyrene secondary standards (J. T. Baker). The standard molar enthalpy of vaporization results at 298.15 K were (77.9 ± 1.4) kJ mol^–1^ for phenanthrene and (86.4 ± 1.4) kJ mol^–1^ for pyrene (Tables S1–S3 in the Supporting Information). The calculated enthalpies are consistent with those reported in the literature.^32^

## Computational Details

### MLR Model

The MLR model is a versatile statistical model for evaluating a continuous target and predictors correlation.^33^

The predictors can be continuous, categorical, or derived fields so that nonlinear relationships are also supported. The model is considered linear because it consists of additive terms, where each term is a predictor which is multiplied by an estimated coefficient (β_*i*_) (see eq 3).

The constant term (intercept, β_0_) is also usually added to the model.^34^ Multiple regression models can be used to predict the value of the dependent variable or to assess the influence that the predictors have on it (the latter should be analyzed with caution so as not to misinterpret the cause-effect).^35^
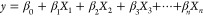
3

The model relates a dependent variable (*y*) with *n* regressor variables (*X*_*n*_) and finally, a random variable (β_0_) that collects all those factors that are not collectable and are associated with chance.^36^

It is important to bear in mind that the magnitude of each partial regression coefficient depends on the units in which the predictor variable is measured, so its magnitude is not associated with each predictor importance. To determine each variable impact over the model, the standardized partial coefficients are used.^37^

### Stochastic Gradient Descent Regression

The SGD algorithm behaves like a straight-line formula, but it is based on a convex function.^38^ The starting point is just an arbitrary point, so the performance can be evaluated. From that consideration, the derivative (or slope) could be determined, the slope is associated with the parameter updates, i.e., weights and bias; at the starting point it will be steeper, but as new parameters are generated, the slope should gradually decrease until it reaches the curve’s lowest point, known as the convergence point.^39^ The SGD runs a training epoch for each example within the data set and updates each parameter of the training example, one at a time.^40^

## Results and Discussion

### Experimental Results

Table 2 shows four experimental results from the compound 7M4MC, which are the data from the purity, the melting point, the enthalpy of phase change, and the heat capacity at constant pressure, including the experimental uncertainties.

**Table 2:** Melting Temperature of 7M4MC^a^

purity	*T*_fus_ (K)	Δ_cr_^l^*H*_m_ (*T*_fus_) kJ mol^–1^	*C*_p,m_ (cr, 298.15 K) J mol^–1^ K^–1^
0.9987	432.9	31.7	
0.9978	433.0	31.9	
0.9976	430.6	29.4	
0.9951	431.2	29.3	
0.9973 ± 0.0015	431.9 ± 1.2	30.6 ± 1.4	1.439 ± 0.023

a The uncertainties correspond to the expanded uncertainty with a level of confidence of 95%, including uncertainty of calibration and *u*(*T*) = 0.1 K. The experiments were made under average atmospheric pressure (78.8 kPa), *u*(*p*) = 1 kPa.

The molar heat capacity was calculated from 273.15 to 388.15 K using the results obtained by DSC, these data are shown in Supporting Information in Table S4; for this calculus, the eq 4 is used, this was obtained from a polynomial regression applied to data in a graph of heat capacity vs temperature.
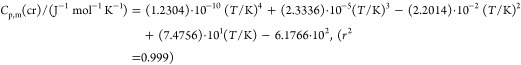
4

On the other hand, Ngoc Toan^41^ determined that the melting temperature ranged from 432 to 435 K, which compared to our value presented a 0.37% error. It is important to mention that in the past decade in the literature, no value for 7M4MC enthalpy of fusion of the compound is reported.

Table 3 shows the 7M4MC combustion results. The six combustions’ complete data sets are shown in the Supporting Information in Table S5.

**Table 3:** Combustion Experiments for 7M4MC at 298.15 K and *p*^o^ = 0.1 MPa

	7M4MC^a^
*m* (7M4MC)/g	0.0408401
*m* (cotton)/g	0.0005911
*m* (platinum)/g	0.2311213
Δ*T*_c_/K	0.891702
ϵ (calor) (−Δ*T*_c_)/kJ	–1.14245
ϵ (cont) (−Δ*T*_c_)/kJ	–0.00111
Δ*U*_ign_/kJ	0.00418
Δ*U*_IBP_/kJ	–1.13938
Δ*U*_corr_/kJ	0.00081
(–*m*Δ_c_*u*^o^)(cotton)/kJ	0.01002
(–*m*Δ_c_*u*^o^) (7M4MC)/kJ	–1.12855
Δ_c_*u*^o^ (7M4MC)/kJ g^–^^1^	–27.6334
average value ⟨−27.6912 ± 0.0162⟩/kJ g^–^^1^	

a *m* is the mass corrected for buoyancy using densities listed in Table 1, Δ*T*_c_ is the corrected temperature rise, ϵ (cont) is the energy equivalent of the contents of the bomb, Δ*U*_ign_ is the ignition energy, and Δ*U*_IBP_ is the energy of the isothermal bomb process, which was calculated by Δ*U*_IBP_ = [ϵ (calor)(−Δ*T*_c_) + ϵ (cont)(−Δ*T*_c_) + (Δ*U*_ign_) (Δ*U*_corr_)]. Δ*U*_corr_ is the correction to standard state and Δ_c_*u*^o^ (7M4MC) is the 7-methoxy-4-methylcoumarin mass energy of combustion. The uncertainty corresponds to the expanded uncertainty with a confidence level of 95%.

The average combustion energy, enthalpy, and uncertainty at 298.15 K and 0.1 MPa are shown in Table 4. To calculate the standard molar enthalpy of formation Δ_f_*H*_m_°(cr) from the molar enthalpy of combustion Δ_c_*H*_m_°(cr) the CO_2_(g) and H_2_O(l) molar enthalpy of formation values were −(393.51 ± 0.13) and −(285.83 ± 0.04) kJ mol^–1^ at 298.15 K, respectively.^42^

**Table 4:** Standard Molar Energy and Enthalpy of Combustion and Standard Molar Enthalpy of Formation in the Solid Phase at 298.15 K

Δ_c_*U*_m_°^a^(kJ mol^–1^)	Δ_c_*H*_m_°^a^(kJ mol^–1^)	Δ_f_*H*_m_°^b^(kJ mol^–1^)
–5266.8 ± 6.2^a^	–5269.3 ± 6.2^a^	–488.5 ± 6.4^b^

a The uncertainties correspond to the expanded uncertainty with a confidence level of 95%, they include the calibration contributions from benzoic acid and energy of combustion of cotton thread.

b The uncertainty corresponds to the expanded uncertainty with a confidence level of 95%, it includes the uncertainties of standard enthalpy of formation of H_2_O (l) and CO_2_ (g).

Table 5 shows the results of vaporization enthalpy for compound 7M4MC at *T*_m_ = 463.15 K (where *T*_m_ is the mean temperature), four series of experiments were performed, and an average of the obtained values is reported (Figures S1–S4 in the Supporting Information).

**Table 5:** Vaporization Enthalpies for 7M4MC

temperature *T*/K	mass *m*/mg	(d*m*/d*t*)·10^9^ kg/s	ν·10^3^ (kg K mol)^1/2^ m^–2^ s^–1^	ln ν	10^3^/*T* K
438.15	1.174	3.630	80.483	–2.520	2.282
443.15	1.164	4.550	101.455	–2.288	2.257
448.15	1.149	5.700	127.813	–2.057	2.231
453.15	1.115	7.040	158.738	–1.840	2.207
458.15	1.109	8.640	195.887	–1.630	2.183
463.15	1.003	10.590	241.404	–1.421	2.159
468.15	0.999	12.800	293.353	–1.226	2.136
473.15	0.988	15.350	353.668	–1.039	2.113
478.15	0.973	18.300	423.858	–0.858	2.091
483.15	0.855	21.680	504.763	–0.684	2.070
488.15	0.723	25.320	592.554	–0.523	2.049

series I			
ln ν = 17.06–8570.44/*T*	*r*^2^ = 0.9994	σ_m_ = 71.09, σ_y_ = 0.15	Δ_l_^g^*H*_m_ (463.15 K)/kJ mol^–1^ = (71.3 ± 0.6)^a^
series II			
ln ν = 16.85–8461.06/*T*	*r*^2^ = 0.999	σ_m_ = 120.77, σ_y_ = 0.26	Δ_l_^g^*H*_m_ (463.15 K)/kJ mol^–1^ = (70.3 ± 1.0)^a^
series III			
ln ν = 16.96–8526.61/*T*	*r*^2^ = 0.9992	σ_m_ = 110.89, σ_y_ = 0.24	Δ_l_^g^*H*_m_ (463.15 K)/kJ mol^–1^ = (70.9 ± 0.9)^a^
series IV			
ln ν = 16.77–8455.23/*T*	*r*^2^ = 0.9997	σ_m_ = 118.56, σ_y_ = 0.26	Δ_l_^g^*H*_m_ (463.15 K)/kJ mol^–1^ = (70.3 ± 1.0)^a^
weighted average: ⟨Δ_l_^g^*H*_m_ (463.15 K) = (70.9 ± 0.8) kJ mol^–1^⟩			

a The uncertainty corresponds to the combined standard and includes the uncertainties of the slope, the rate of mass loss, and the temperature.

Table 6 contains the enthalpy of sublimation calculation at 298.15 K, in addition this table presents the results for the enthalpy of fusion and vaporization under experimental conditions. The pertinent adjustment to 298.15 K was determined by applying eqs 5–7.^43,44^
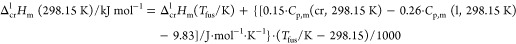
5
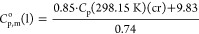
6
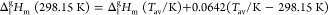
7

**Table 6:** Determination of Enthalpy of Sublimation at 298.15 K^a^

Δ_cr_^l^*H*_m_ (298.15 K)^b^ (kJ mol^–^^1^)	Δ_l_^g^*H*_m_ (298.15 K)^c^ (kJ mol^–^^1^)	Δ_cr_^l^*H*_m_ (298.15 K) + Δ_l_^g^*H*_m_ (298.15 K) (kJ mol^–^^1^)
TGA		
23.4 ± 1.4	81.5 ± 1.6	104.9 ± 2.1

a All the uncertainties correspond to twice the combined standard.

b Value calculated from eqs 5 and 6.

c Value calculated from eq 7.

The enthalpy of sublimation was calculated by adding the enthalpy of vaporization and the enthalpy of fusion at 298.15 K. The enthalpy of sublimation for this compound has not been reported elsewhere yet.

Meanwhile, the standard molar enthalpy of formation in the gas phase was obtained from the standard molar enthalpy of formation in the crystalline phase plus the enthalpy of sublimation; as seen in Table 7.

**Table 7:** Standard Molar Enthalpies of Formation and Sublimation of 7M4MC at 298.15 K^a^

Δ_f_*H*_m_^o^(cr) (kJ mol^–^^1^)	Δ_cr_^g^*H*_m_ (298.15 K) (kJ mol^–^^1^)	Δ_f_*H*_m_^o^(g) (kJ mol^–^^1^)
–488.5 ± 6.4	104.9 ± 2.1	–383.6 ± 6.7

a The uncertainty corresponds to the expanded uncertainty with a level of confidence of 95%.

### Theoretical Results

For assessing the precision from those values obtained experimentally, machine learning was used. To predict the 7M4MC enthalpy of formation in the gas phase, a data set was created based on the functional groups separation proposed by Benson;^23^ for these analysis, the ester family compounds were considered because this is the main functional group presented in coumarins.

**Figure 2:**
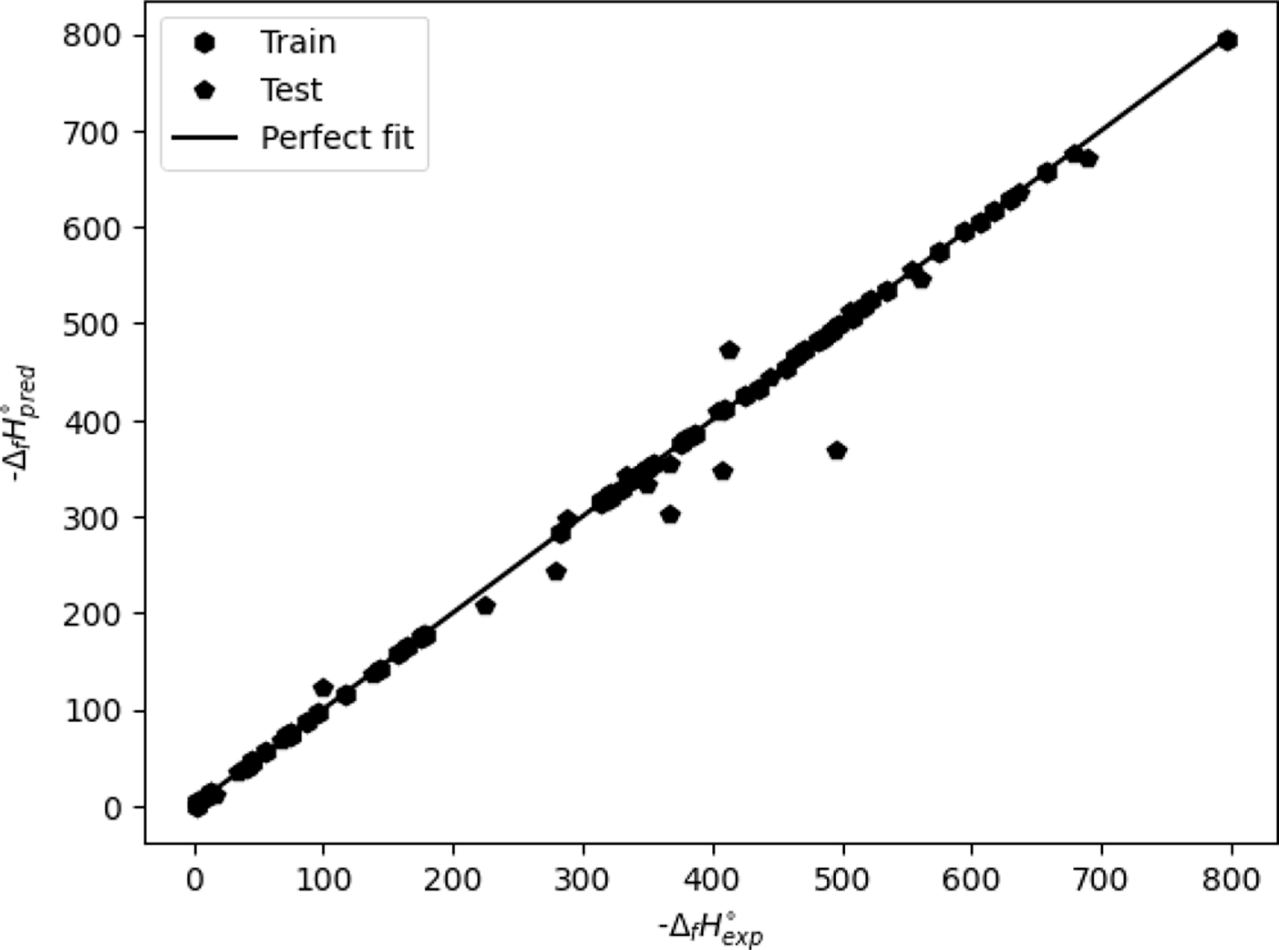
Experimental and predicted value comparison from −Δ_f_*H*_m_° (g, 298.15 K) by MLR.

From a literature review, a data set of 84 experimental values was obtained, and the data was separated into training and testing using the hold out model (70/30) and the seed 204, respectively; so as a result, the values obtained in this work can be reproducible. The metrics results are shown in Table 8, likewise Figure 2 presents the comparison between the experimental and predicted value for all compounds as well as the linear regression is included as a perfect fit.

**Table 8:** Algorithm Evaluation Metrics

MLR								
	train	test		train	test		train	test
*R*^2^	0.9999	0.9669	MAE^a^	0.8827	18.8324	RMSE^b^	1.2825	34.0633

SGD								
	train	test		train	test		train	test
*R*^2^	0.9998	0.9658	MAE^a^	2.0079	19.4297	RMSE^b^	2.5765	34.6121

a Mean absolute error.

b Root mean squared error.

The evaluation metrics in the test set are low due to the fact that the data set does not consider the esters’ aromatic interaction; thus, not enough experimental values were found, and as a result an addition of 24 aromatic compounds was permitted to compensate for the precision. The result after that consideration was favorable, as is shown in Table 9, where the standard molar enthalpies of formation in the gas phase exclusively for the coumarins are reported; furthermore, a Δ parameter was added, which represents the error between the experimental and predicted values.

**Table 9:** Comparison between Literature and Predicted Values of −Δ_f_*H*_m_° (g, 298.15 K) in kJ mol^–1^ and Benson

compound	literature	predicted^a^	Δ	predicted^b^	Δ	Benson^c^	Δ
coumarin	176.8^d^	176.8	0	179.4	2.6	173.7	–3.1
3-hydroxycoumarin	367.7^e^	353.6	–14.1	354.3	–13.4	371.1	3.4
4-hydroxycoumarin	351.4^e^	349.5	–1.9	350.7	–0.7	371.1	19.7
5-hydroxycoumarin	344.9^f^	345.4	0.5	347.0	2.1	371.1	24.6
6-hydroxycoumarin	339.8^g^	341.3	1.5	343.4	3.6	371.1	31.3
7-hydroxycoumarin	337.5^f^	337.2	–0.3	339.7	2.2	371.1	33.6
8-hydroxycoumarin	349.2^f^	333.0	–16.2	336.0	–13.2	371.1	21.9
5-methoxycoumarin	329.7^h^	327.9	–1.8	329.2	–0.5	327.1	–2.6
6-methoxycoumarin	321.5^h^	323.8	2.3	325.5	4.0	327.1	5.6
7-methoxycoumarin	321.6^h^	319.7	–1.9	321.9	0.3	327.1	5.5
8-methoxycoumarin	314.2^h^	315.6	1.4	318.2	4.0	327.1	12.9
7M4MC	383.6^i^	384.9	–1.3	382.2	1.4	415.5	31.9

a Values predicted using MLR.

b Values predicted using the SGD regression.

c Values calculated using Benson.

d Taken of ref (45).

e Taken of ref (46).

f Taken of ref (47).

g Taken of ref (48).

h Taken of ref (49).

i Experimental value of this work.

As observed in Table 9, the MLR and SGD values are quite close to the experimental value, so it means that the proposed models can be applied to predict the compound of interest’s enthalpies.

By analyzing the coumarin behavior and the two regression approach, we have observed that those theoretical tools are useful to predict the enthalpy of a desired compound because the difference between the two methods has a similar variation with respect to the experimental value.

**Figure 3:**
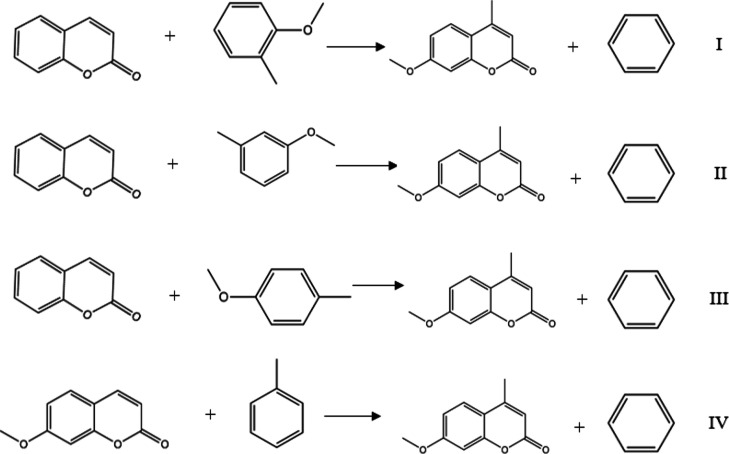
Homodesmic reactions used in the 7M4MC enthalpy of formation determination.

On the other hand, using the Benson’s method results in a greater error due to the lack of updating of the data and by not considering the difference in between isomers, it falls into the same result for different kinds of molecules. Another theoretical method to obtain the enthalpy of formation in the gas phase is through the use of homodesmic reactions and, thus, is necessary to propose the 7M4MC reactions, as shown in Figure 3.

The molecules proposed in reactions I, II, III, and IV were predicted by MLR. Besides for the ester compounds, a data set was constructed using 78 gas phase enthalpy of formation values in total; however, for the aromatic compounds, 53 values were used; in both cases, a hold-out (70/30) was maintained.

The seeds used were 39 and 508 for each type of compound, respectively (evaluation metrics are shown in Table S6 in the Supporting Information and Figures S5 and S6).

Table 10 shows the prediction results from those molecules presented in Figure 3. However, in Table 11, the homodesmic reaction results are presented, and the difference between the 7M4MC predicted value against the experimental value for each homodesmic reaction is given in brackets.

**Table 10:** Theoretical Results for the Molecules Used in Homodesmic Reactions

compound	Δ_f_*H*_exp_°(g, 298.15 K)	Δ_f_*H*_pred_°(g, 298.15 K)	Δ
coumarin	–176.8^a^	–176.8	0.0
1-methoxy-2-methylbenzene	–106.6 ± 1.6^b^	–109.5	–2.9
1-methoxy-3-methylbenzene	–102.6 ± 5.0^b^	–100.6	2.0
1-methoxy-4-methylbenzene	–99.0 ± 2.0^b^	–98.4	0.6
7-methoxycoumarin	–321.6 ± 2.8^c^	–319.7	1.9
benzene	82.9 ± 0.9^d^	83.5	–0.6
toluene	50.1 ± 1.1^d^	49.3	0.8
7M4MC	–383.6 ± 6.7^e^	–384.9	–1.3

a Taken of ref (45).

b Taken of ref (51).

c Taken of ref (49).

d Taken of ref (52).

e Experimental value of this work.

**Table 11:** Computational Estimates of the Standard Enthalpy of Formation in the Gas Phase at 298.15 K of the 7M4MC

	*R*	–Δ_f_*H*_pred_°(g)/(kJ mol^–1^)	–exp
7M4MC	I	381.3 (–2.1)	383.6 ± 6.7
	II	386.2 (2.6)	
	III	384.8 (1.2)	
	IV	385.4 (1.8)	

As observed from those results reported in Table 11, it shows that although all the proposed reactions values are close to the experimental value, the best reaction is III where the 1-methoxy-4-methylbenzene compound is presented. From this analysis, it can be suggested that the use of MLR to predict the enthalpy of formation of organic compounds is fast and reliable to the conventional software already used.^50^

Although one of the purposes of SGD is to improve the coefficients presented for MLR, we observed that similar results are obtained with both MLR and SGD, so the application of MLR is also a trustable option to be applied in these thermochemical property prediction.

To estimate the enthalpy of formation in the crystalline phase, a conventional regression was performed based on the coumarins reported experimentally in this phase because for esters and aromatics the reported condensed phase is the liquid phase. The regressors considered were the amount of C, H, and O atoms together and *X*_3_ and *X*_4_ as variables, which indicates where each of the coumarin radicals used binds, as seen in eq 8. The coefficient of determination (*R*^2^) was 0.9951. The resultant predictions are listed in Table 12.
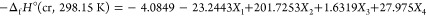
8where *X*_1_ represents the number of H atoms, *X*_2_ is the number of O atoms, *X*_3_ is the radical 1, and *X*_4_ is the radical 2. To identify the radical position, the numbering must begin from the carbonyl group toward the methoxy group, as shown in Figure 1.

**Table 12:** Comparison between Experimental and Predicted Values of −Δ_f_*H*°(cr, 298.15 K) Using the Regression in kJ mol^–1^

compound^a^	experimental	predicted	Δ
C^b^	259.9	259.9	0
7H4MC^c^	540.8	538.5	–2.3
6M4MC^c^	488	490.3	2.3
6HC^d^	466.2	471.4	5.2
7HC^e^	471.1	473.0	1.9
3HC^f^	459.6	466.5	6.9
4HC^f^	479.9	468.2	–11.7
7MC^g^	428.9	426.6	–2.3
7M4MC^h^	488.5	492.0	3.5

a Compound: coumarin (C); 7-hydroxy-4-methylcoumarin (7H4MC); 6-methoxy-4-methylcoumarin (6M4MC); 6-hydroxycoumarin (6HC); 7-hydroxycoumarin (7HC); 3-hydroxycoumarin (3HC); 4-hydroxycoumarin (4HC); and 7-methoxycoumarin (7MC).

b Taken of ref (23).

c Taken of ref (53).

d Taken of ref (48).

e Taken of ref (47).

f Taken of ref (46).

g Taken of ref (49).

h Experimental value of this work.

As can be seen from eq 8, the carbon atoms amount within the compounds does not affect the enthalpy of formation in the crystalline phase estimation, the resultant value for compound 7M4MC was −(492.0 ± 4.1) kJ mol^–1^ (the uncertainty represents the average absolute error of the coumarins presented in Table 12), this value has a difference of 3.5 kJ mol^–1^ with respect to the experimentally obtained.

The predicted values of coumarins using this regression are quite close to those reported in the literature, so it is a good option to perform this type of analysis when few data are available, and it is necessary to compare with an experimental value.

Finally, an additional advantage for MLR is that it is possible to obtain regression coefficients; these coefficients represent a change and update to the conventional ones shown by Benson, as it is shown in Table 13.

**Table 13:** Update of Benson Functional Groups of −Δ_f_*H*_m_° (g, 298.15 K) for Esters and Coumarins in kJ mol^–1^

group	value	group	value
CO–(C_*D*_)(O)	11.32	O–(C_*B*_)(CO)	28.56
CO–(C)(O)	25.79	C_*D*_–(O)(H)	0
CO–(O)(C_*B*_)	24.17	C–(H)_2_(CO)_2_	127.97
O–(H)(CO)	0	C–(H)_3_(CO)	75.37
O–(C)(C_*B*_)	–12.55	C–(H)_2_(CO)(C_*D*_)	–3.29
O–(H)(C_*B*_)	86.57	C–(H)_2_(CO)(C_*T*_)	0
C_*D*_–(H)(CO)	3.71	C–(O)(C)_3_	–54.98
C_*D*_–(C)(CO)	7.61	C–(H)(O)(C)_2_	–8.64
C_*B*_–(CO)(C_*B*_)_2_	36.31	C–(H)_2_(O)(C)	32.42
C_*B*_–(O)(C_*B*_)_2_	102.58	O–(CO)(O)	0
C–(CO)(C)_3_	–202.02	C_*D*_–(H)(C)	6.12
C–(H)(CO)(C)_2_	–23.98	C_*D*_–(H)(C_*D*_)	0
C–(H)_2_(CO)(C)	14.58	C–(H)_2_(C)(C_*D*_)	5.70
C–(H)_3_(O)	82.38	C_*T*_–(C)	0
C–(H)_3_(C_*D*_)	11.32	C_*B*_–(C_*B*_)_3_	22.86
C–(H)_3_(C)	82.40	C_*B*_–(H)(CO)(C_*B*_)	–12.55
C–(H)_3_(C_*B*_)	41.51	C_*B*_–(CO)_2_(C_*B*_)	0
C–(H)_2_(C)_2_	20.33	rsc	–17.59
C–(H)(C)_3_	–9.91	radical 1	–4.11
C_*D*_–(H)_2_	16.60	radical 2	–1.86
C_*B*_–(H)(C_*B*_)_2_	–11.82	radical 3	0.68
C_*B*_–(C)(C_*B*_)_2_	–6.02	correction O-	–10.37
C–(H)_2_(C)(C_*B*_)	–20.83	correction M-	–4.82
CH_3_(tert)	2.46	correction P-	–4.09
CH_3_(qua)	0	group aromatic	–57.16
CO–(C)(CO)	81.83	group coumarin	22.86
CO–(C)(C_*B*_)	35.00	group ester	34.30
O–(C_*D*_)(CO)	0	O–(C)(CO)	158.54
*H*_0_ = 34.86^a^			

a Represents the β_0_ value.

## Conclusions

The enthalpy of fusion was determined by DSC and the enthalpy of vaporization was obtained by thermogravimetric analysis. The experimental standard molar enthalpy of formation in the gas phase of 7M4MC as a result from the standard molar enthalpy of formation in the solid phase and the standard molar enthalpy of sublimation resulting in −383.6 kJ mol^–1^, this value represents an excellent agreement concerning the value predicted from machine learning algorithms, which have a difference of 1.3 kJ mol^–1^ with respect to MLR and 1.4 kJ mol^–1^ with respect to the SGD regression. Based on that obtained from the experimental section, the enthalpy of formation in the crystalline phase was predicted using a fitting equation that was able to distinguish between the structural isomerism on different compounds and although it was only applied to a small data set, it was possible to demonstrate a prediction path when limited experimental values are available. Finally by using the fitting equation, a difference of 3.5 kJ mol^–1^ was obtained, and with the homodesmic reactions it was possible to propose an alternative method capable of predicting the enthalpy of formation in the gas phase; thus, the optimal reaction had a difference with an experimental value of 1.2 kJ mol^–1^ and the biggest difference of 2.6 kJ mol^–1^ with respect to the experimental value.

## Supporting Information Available

Data set consisting of values obtained experimentally, MLR and SGD, 39 for the set with ethers and 508 for the aromatic compounds (ZIP)
