# Supplementary figures and images for: 7-Methoxy-4-methylcoumarin: Standard Molar Enthalpy of Formation Prediction in the Gas Phase Using Machine Learning and Its Comparison to the Experimental Data
Source: ACS Omega. 2023 Dec 14;8(51):49037–45. doi: 10.1021/acsomega.3c06756 (PMC10753555; doi:10.1021/acsomega.3c06756)

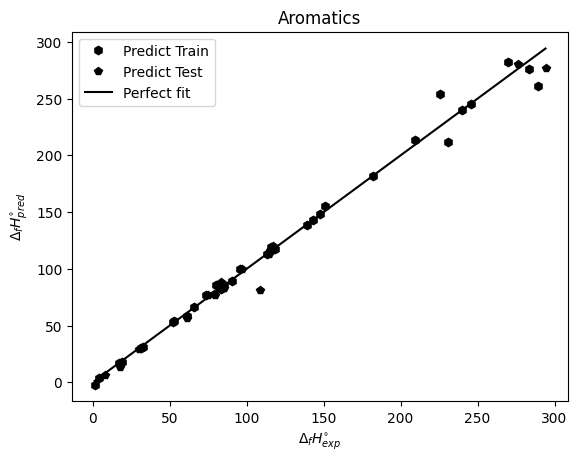

Supplement: Supplementary file 1 — ao3c06756_si_001.zip [file ao3c06756_si_001.zip › Aromatics.png]

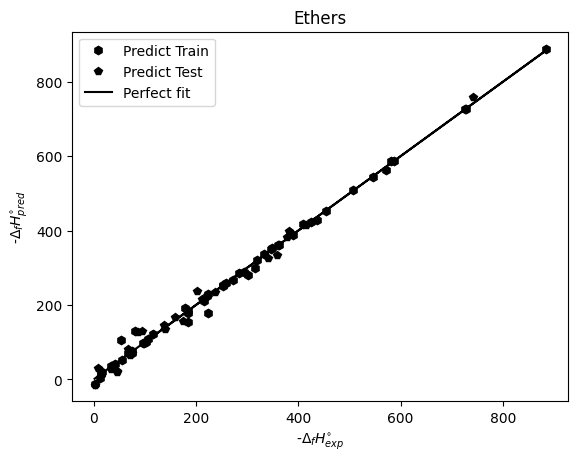

Supplement: Supplementary file 1 — ao3c06756_si_001.zip [file ao3c06756_si_001.zip › Ethers.png]

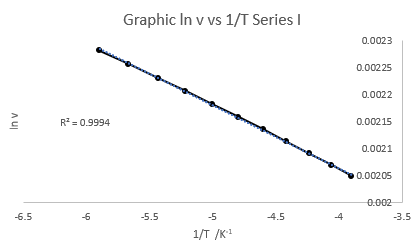

Supplement: Supplementary file 1 — ao3c06756_si_001.zip [file ao3c06756_si_001.zip › Series 1 TGA.png]

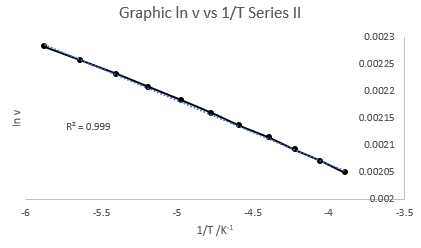

Supplement: Supplementary file 1 — ao3c06756_si_001.zip [file ao3c06756_si_001.zip › Series 2 TGA.png]

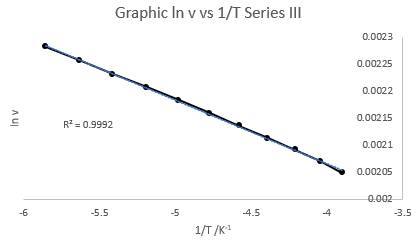

Supplement: Supplementary file 1 — ao3c06756_si_001.zip [file ao3c06756_si_001.zip › Series 3 TGA.png]

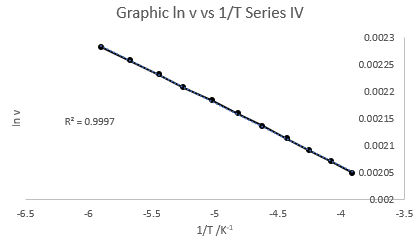

Supplement: Supplementary file 1 — ao3c06756_si_001.zip [file ao3c06756_si_001.zip › Series 4 TGA.png]
